# Supplementary material for: Anti-HIV-1 HSPC-based gene therapy with safety kill switch to defend against and attack HIV-1 infection
Source: Mol Ther Methods Clin Dev. 2025 May 15;33(2):101486. doi: 10.1016/j.omtm.2025.101486 (PMC12152878; doi:10.1016/j.omtm.2025.101486)
Supplement: Document S1. Figures S1–S14 [file mmc1.pdf]

OMTM, Volume *33*

Supplemental information

Anti-HIV-1 HSPC-based gene therapy with safety kill switch to defend against and attack HIV-1 infection

Qi Guo, Keval Parikh, Jian Zhang, Alexander Brinkley, Grace Chen, Natnicha Jakramonpreeya, Anjie Zhen, and Dong Sung An

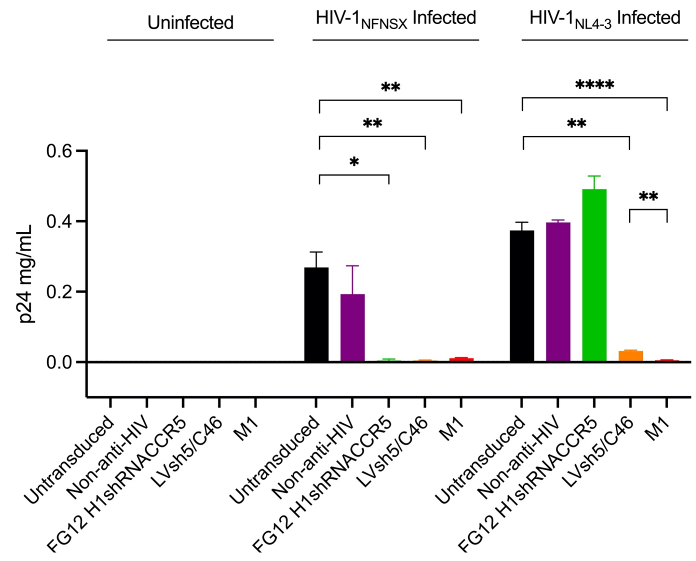

Figure S1. *In-vitro* HIV-1 inhibition by M1 vector compared to mono CCR5sh1005 (FG12H1shRNACCR5) or dual CCR5sh1005 and C46 (LVsh5/C46) anti-HIV-1 lentiviral vectors. Untransduced and non-anti-HIV vector-transduced MT4-CCR5 cells were used as negative controls. MT4-CCR5 cells were challenged with R5 tropic HIV-1_{NFNSX} (MOI 1) or X4 tropic HIV-1_{NL4-3} (MOI 0.01) virus. p24 capsid protein levels in cell culture supernatant were determined by p24 ELISA assay 7 days post HIV-1 challenge and used to assess inhibition ability. Data shows results from three independent experiments. Bars and error bars show Mean ± S.E.M, respectively. Student's t-test was performed to calculate significance. *$p<0.05$, **$p<0.01$, and ****$p<0.0001$.

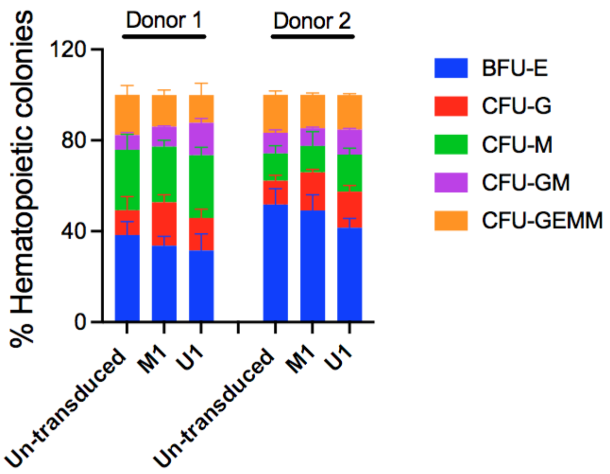

Figure S2. Colony-forming units (CFUs) of untransduced, M1, and U1 vector-transduced FL-CD34+ cells after 14 days of culture. % of burst-forming unit-erythroid (BFU-E), colony-forming unit-granulocytes/macrophages (CFU-G, CFU-M, CFU-GM), and multipotential progenitor cells (CFU-GEMM) = 100 × (each colony type CFU counted/total CFU counted). Data were collected from triplicates from two independent donors.

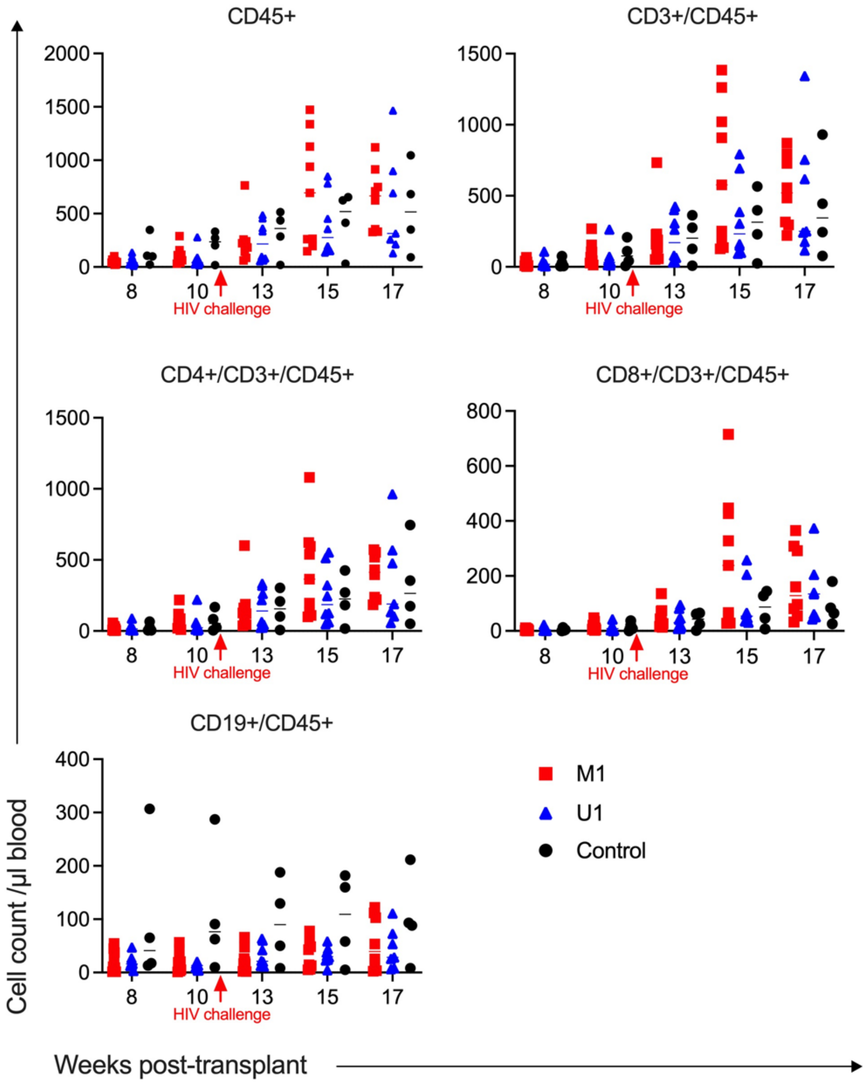

Figure S3. Human multilineage hematopoietic cell reconstitution in peripheral blood of individual mouse from 8 weeks post-vector transduced HSPC transplant (line graphs are shown in Figure 2C). Cell surface markers of human lymphocytes (CD45), T-cells (CD3, CD4, and CD8) and B-cells (CD19) were determined by mAb staining and flow cytometry.

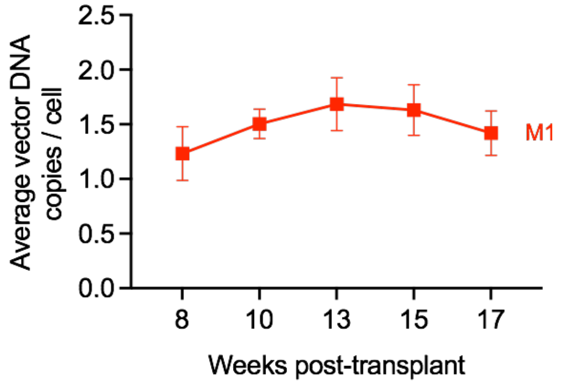

Figure S4. Vector copy number data in replicate (donor 2) huBLT mouse experiment with M1 vector-transduced HSPC transplanted huBLT mice. Vector-marking levels were determined in peripheral blood cells from 8 weeks to 17 weeks post-transplant by digital PCR. Average vector DNA copies were calculated by VCN = [WPRE DNA copies in vector DNA/ul]/[human β-globin copies/ul/2]. Dots and error bars show Mean ± S.E.M, respectively.

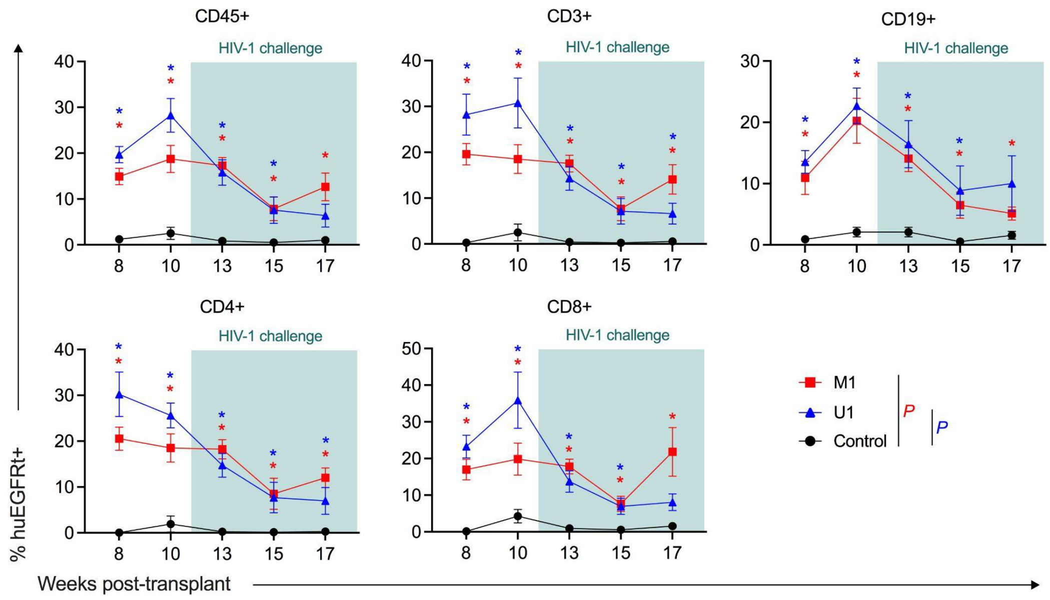

Figure S5. Percentage of huEGFRt expressing cells (huEGFRt+) in human hematopoietic lineages of peripheral blood in HSPC transplanted huBLT mice. Dots and error bars show Mean ± S.E.M, respectively. Mann-Whitney U test was performed to calculate significance, *p <0.05.

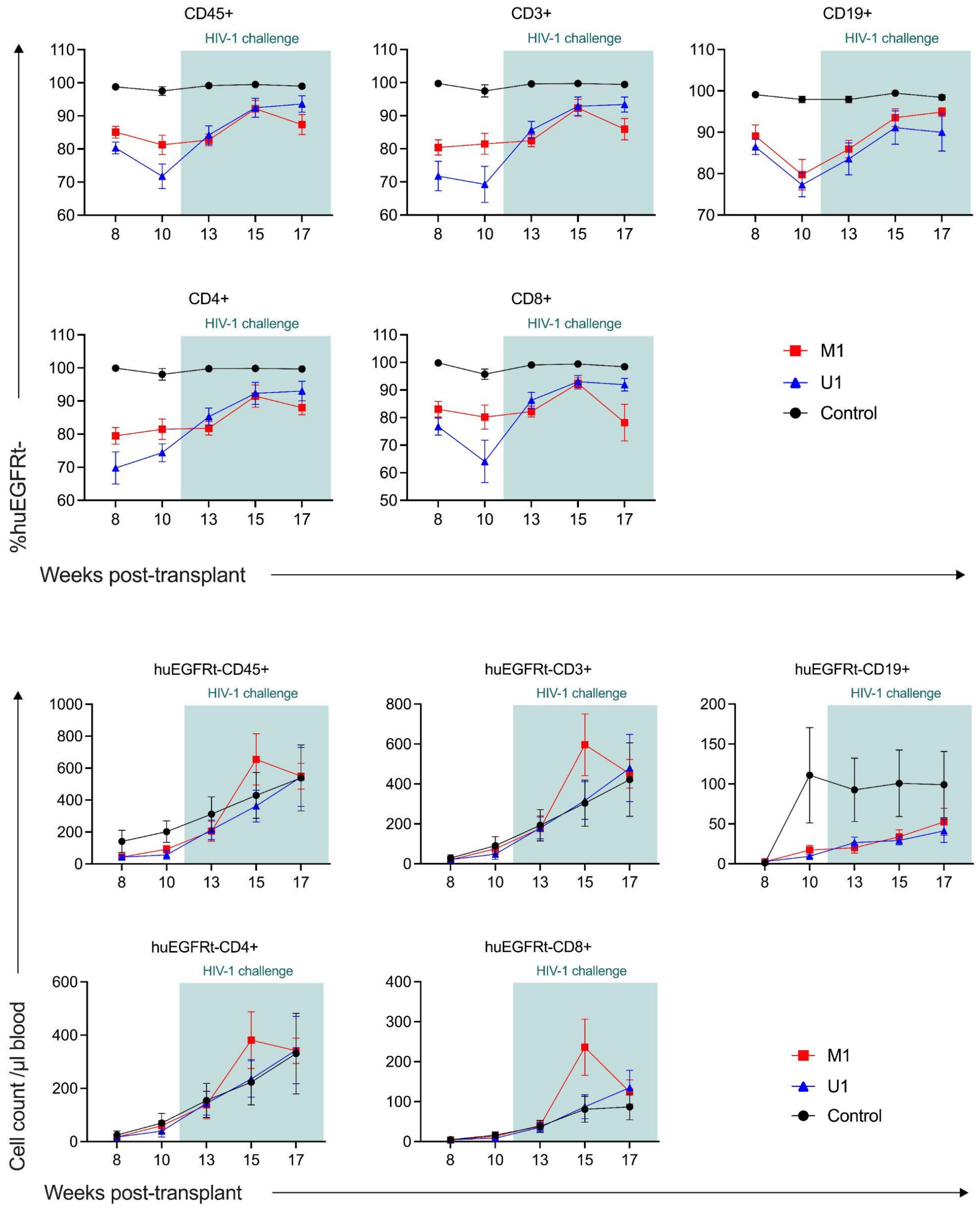

Figure S6. Percentage and absolute cell count/µL of huEGFRt non-expressing cells (huEGFRt-) in human hematopoietic lineages of peripheral blood in HSPC transplanted huBLT mice. Dots and error show Mean ± S.E.M, respectively.

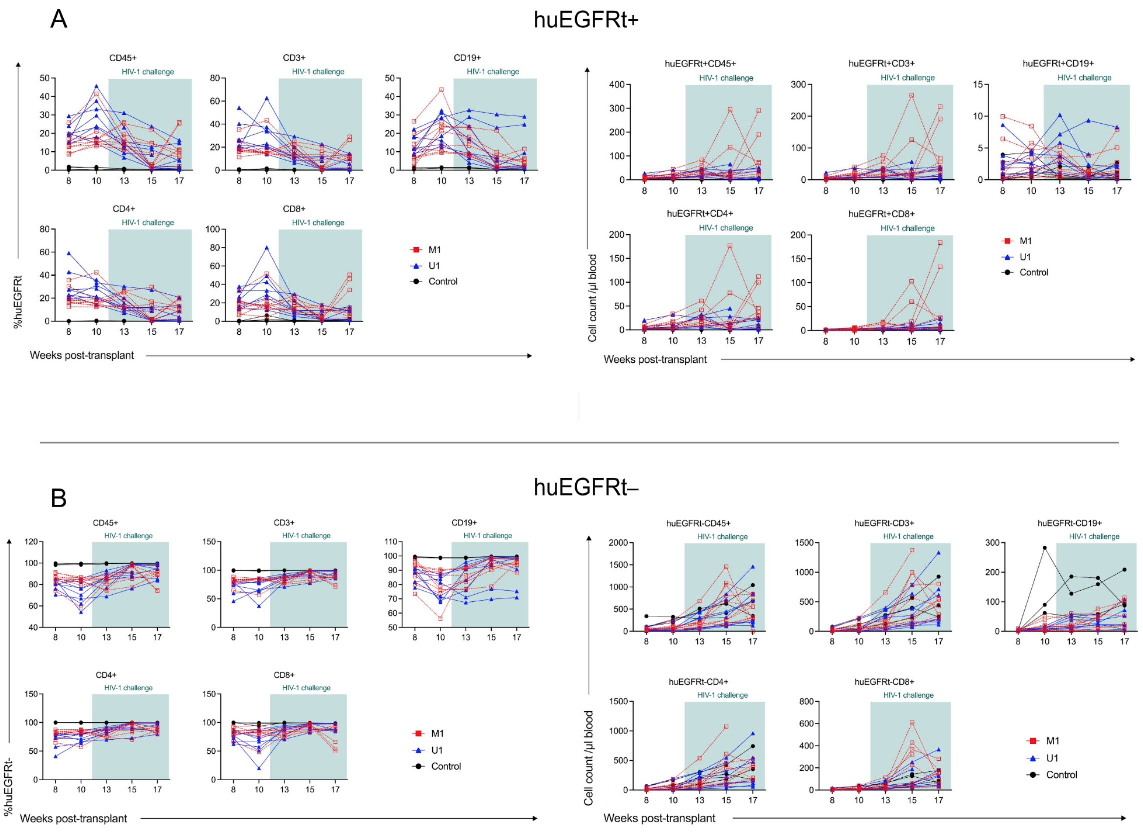

Figure S7. (A) Longitudinal gene marking (huEGFRt+) or (B) huEGFRt- marking in percentages and cell count/μL of peripheral blood in human multilineage hematopoietic populations from individual mice. Each dot represents an individual mouse from the corresponding group.

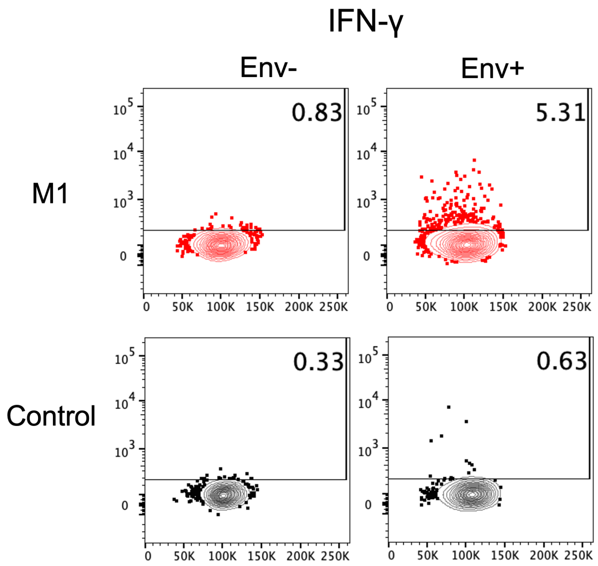

Figure S8. Representative *ex vivo* cytokine production from vector-transduced CD8+ T splenocytes. Effector cells from vector transduced HPSC transplanted huBLT mice were co-cultured with Env+ target cells (PMA/ionomycin activated ACH2 cells) or unstimulated Env- cells (medium only) as a negative control *ex vivo*. Data was collected from our replicate huBLT mice experiment (donor 2). Cells were collected at time of mouse necropsy (week 20 post-transplant) and cytokine production was measured by flow cytometry.

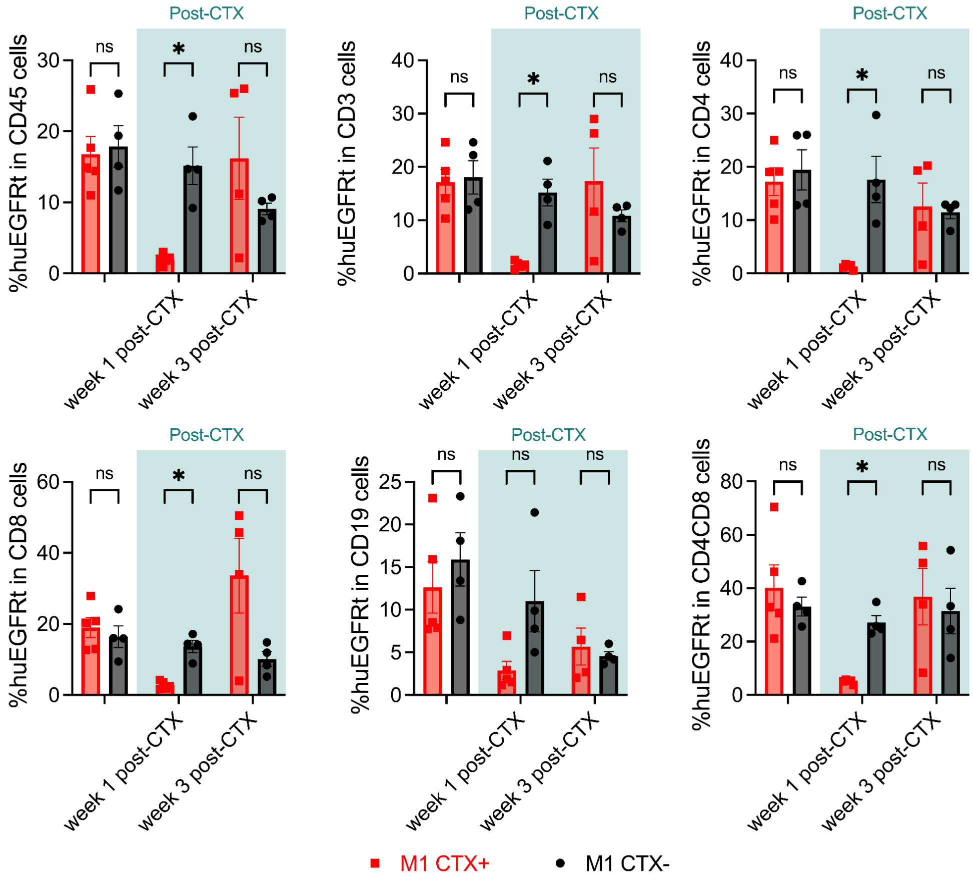

Figure S9. HuEGFRt expression level in multilineage human lymphocytes (CD45), T-cells (CD3, CD4, and CD8) and B-cells (CD19) in CTX treated and untreated huBLT mice that were not infused with human NK cells. Blood was collected 1 week before CTX treatment, and 1 and 3 weeks post-onset of CTX treatment. HuEGFRt expression was measured by flow cytometry using mAb CTX-PE. Dots and error bars show Mean ± S.E.M, respectively. Mann-Whitney U test was performed to calculate significance. NS = not significant, and *p <0.05.

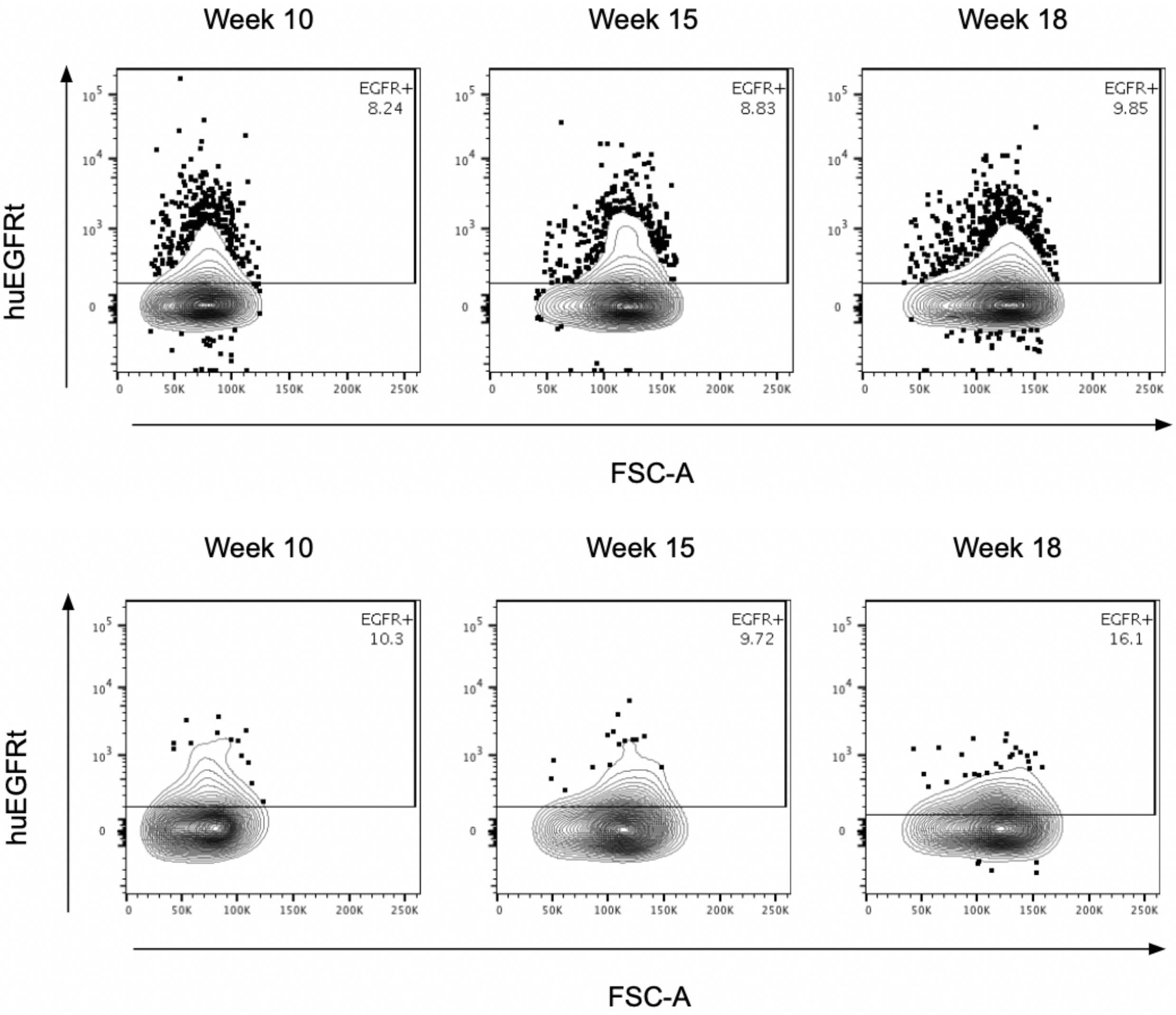

Figure S10. Representative longitudinal flow cytometry plots showing huEGFRt expression in peripheral blood of a CTX-untreated mouse. Peripheral blood was collected at 10, 15, and 18 weeks post-transplant. Top panel shows percent huEGFRt expression within human CD45+ lymphocytes. Bottom panel shows percent huEGFRt expression within human CD45/CD3/CD8+ T cells. FSC-A: Forward Scatter Area.

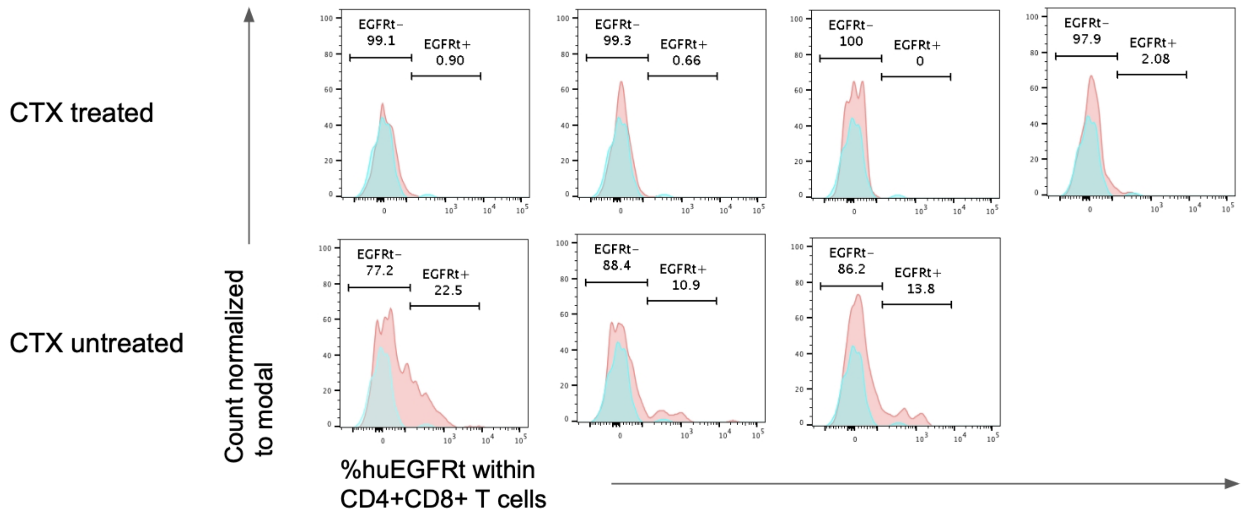

Figure S11. Percentage expression of huEGFRt+ vs huEGFRt- cells within CD4+CD8+ T cell population of splenocytes collected from CTX treated and untreated M1 huBLT mice (as shown in Figure 3D). HuEGFRt expression was measured by flow cytometry using mAb CTX-PE. Each plot represents an individual mouse of the treatment group. Blue plot represents a single untransduced, untreated huBLT mouse used as a negative control.

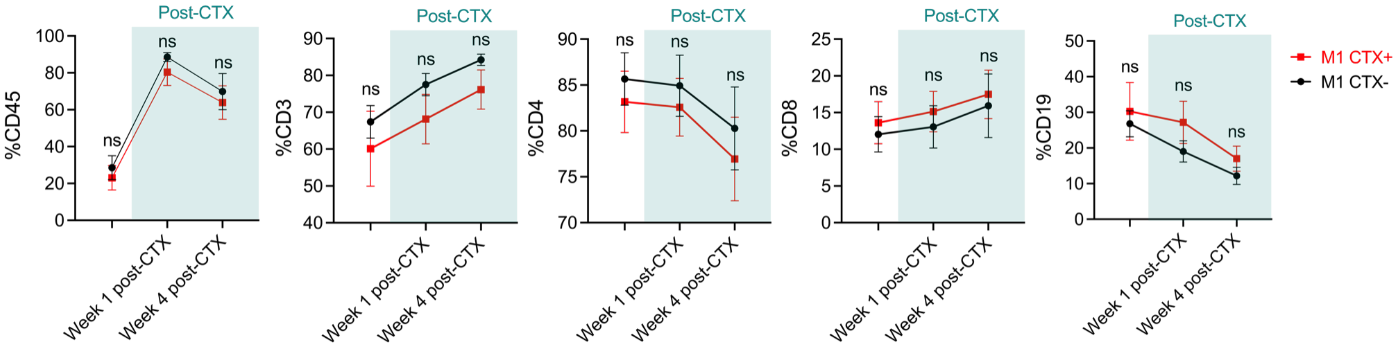

Figure S12. Human multilineage hematopoietic cell reconstitution in peripheral blood of CTX treated and untreated huBLT mice with huEGFRt+ cell lineages. Blood samples were collected at 3 weeks before CTX treatment, and 1 and 4 weeks post-treatment. Surface markers of human lymphocytes (CD45), T-cells (CD3, CD4, and CD8) and B-cells (CD19) were stained and measured by flow cytometry.

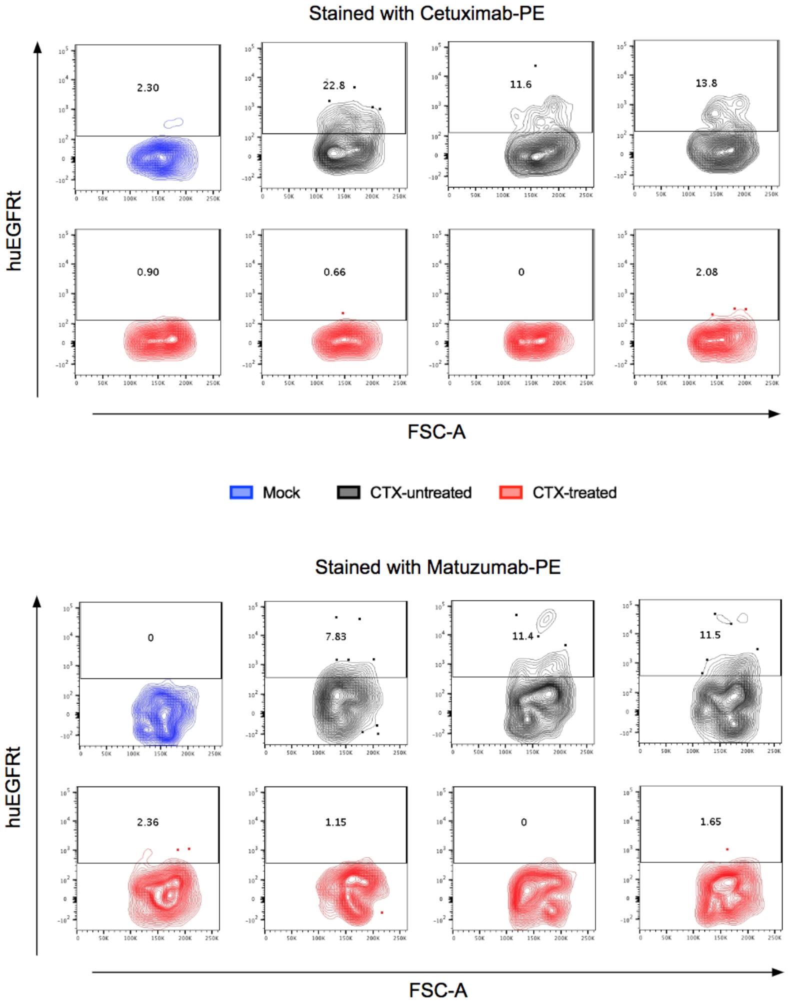

Figure S13. Flow cytometry plots showing percent huEGFRt expression within CD4+CD8+ T cell population of splenocytes. Each plot represents an individual mouse from the corresponding group. "CTX-untreated" splenocytes (black) were from CTX untreated M1 huBLT mice. "CTX-treated" splenocytes (red) were from CTX treated M1 huBLT mice. "Mock" splenocytes (blue) were from untransduced, untreated huBLT mice, serving as a negative control. Splenocytes were stained with two different mAbs, CTX-PE and non-competing anti-huEGFR mAb MTZ-PE. MTZ-PE was utilized to confirm the depletion of huEGFRt expressing population. Expression was measured by flow cytometry. FSC-A: Forward Scatter Area.

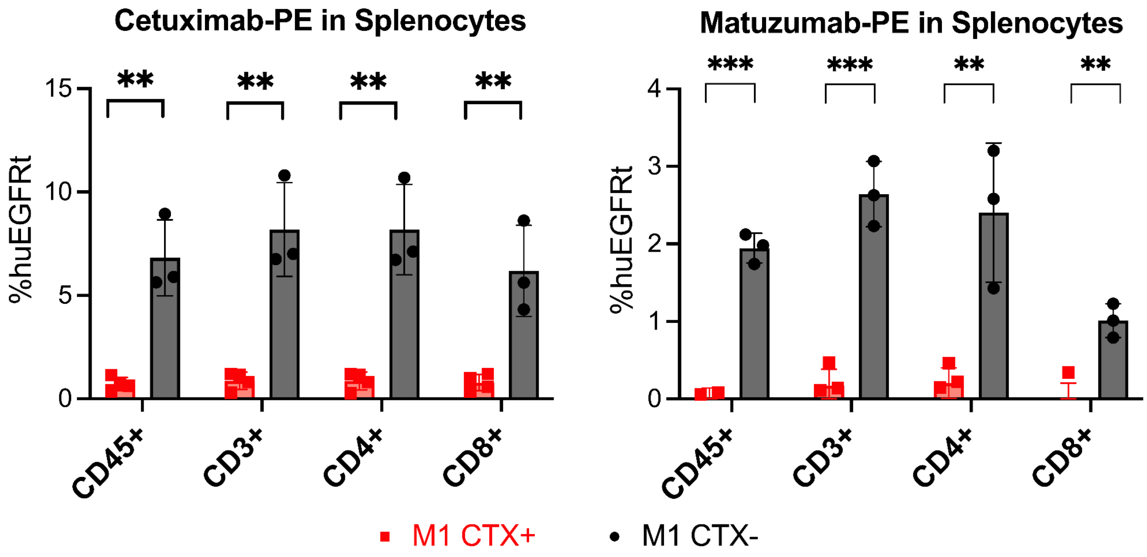

Figure S14. HuEGFRt expression level across multilineage human cell populations including human lymphocytes (CD45), T-cells (CD3, CD4, and CD8) and B-cells (CD19) in splenocytes collected from CTX-treated and untreated huBLT mice. Samples were stained with two different mAbs CTX-PE (as shown in Figure 3C) and non-competing anti-huEGFR mAb MTZ-PE. MTZ-PE was utilized to confirm depletion of huEGFRt expressing population, and measured by flow cytometry. Dots and error bars show Mean ± S.E.M, respectively. Student's t-test was performed to calculate significance. **p <0.01, and ***p <0.001.
